# Association between circulating ω-3 polyunsaturated fatty acid levels and left cardiac myocardial strain in hypertension: a CMR-FT study

**DOI:** 10.3389/fnut.2026.1797442

**Published:** 2026-04-14

**Authors:** Linzhuo Cui, Aoran Xing, Ming Liu, Ting Wei, Jianbo Lyu, Xiaofeng Qu

**Affiliations:** Department of Radiology, The Second Affiliated Hospital of Dalian Medical University, Dalian, Liaoning, China

**Keywords:** CMR-FT, hypertension, myocardial strain, sex differences, ω-3 polyunsaturated fatty acids

## Abstract

**Objective:**

To assess the association between circulating polyunsaturated fatty acids (PUFAs) and left cardiac myocardial strain in hypertensive patients using cardiac magnetic resonance feature tracking.

**Methods:**

This retrospective study included 364 patients with preserved left ventricular ejection fraction (179 hypertensive, 185 controls). Left atrial (LA) and left ventricular (LV) strain parameters were derived from CMR images using CVI42 software. Group comparisons were performed using independent t-tests, ANCOVA, Mann–Whitney U tests, or chi-square tests as appropriate. Univariate and multivariable linear regression were used to examine associations between ω-3 PUFA levels and myocardial strain parameters. Logistic regression and ROC curve analysis were conducted to evaluate predictive value. Inter-observer agreement was assessed using intraclass correlation coefficients.

**Results:**

After adjustment for age, sex, BMI, blood pressure, triglycerides, and creatinine, no significant differences in ω-3 PUFA levels or their subtypes were observed between groups (all *p* > 0.05). In hypertensive patients, docosapentaenoic acid (DPA), docosahexaenoic acid (DHA), and eicosapentaenoic acid (EPA) were independently and negatively associated with multiple LV strain parameters, including mid-wall longitudinal strain, global radial strain, mid-wall radial strain, mid-wall circumferential strain, and apical longitudinal strain, as well as LA functional parameters, including reservoir strain and strain rate (all *p* < 0.05). Sex differences were noted: only DHA was negatively associated with apical radial strain in males, whereas DHA, DPA, and EPA were negatively associated with multiple LV basal and global strain parameters in females (all *p* < 0.05). ROC analysis showed DHA and DPA had moderate predictive value for LV dysfunction (AUC 0.613, 0.602), while DHA, EPA, and total ω-3 predicted LA dysfunction (AUC 0.612, 0.609, 0.629).

**Conclusion:**

Blood ω-3 PUFA levels in hypertensive patients are independently associated with impaired left cardiac myocardial strain and increased dysfunction risk, an effect observed only in the hypertensive group. Different ω-3 subtypes demonstrate predictive value for left cardiac dysfunction. LV dysfunction is primarily associated with DHA and DPA, while LA dysfunction with EPA and total ω-3. Female patients are more sensitive to this effect. Blind ω-3 supplementation should be avoided, and sex-specific management strategies considered.

## 1 Introduction

Cardiovascular diseases (CVDs) have emerged as the leading global health threat in terms of mortality and disability. In China, the annual number of reported CVDs deaths significantly exceeds that of India, Russia, and the United States, ranking first worldwide (1). Given the severe epidemic status of CVDs in China, it is particularly urgent to enhance preventive and therapeutic measures. Abnormal fatty acid metabolism is closely associated with a range of cardiovascular diseases, including atherosclerosis, myocardial infarction, and heart failure (HF). The heart relies on the generation and utilization of adenosine triphosphate (ATP) to sustain its basal metabolism and function, with approximately 80% of cardiac ATP being produced through mitochondrial oxidative phosphorylation, primarily via fatty acid oxidation (FAO). Under pathological conditions, FAO decreases while glucose utilization increases, leading to a reduction in energy production efficiency (2). Studies have confirmed that reducing the accumulation of lipotoxic substances within the myocardium can mitigate their adverse effects on myocardial metabolism, thereby facilitating the recovery of cardiomyocyte energy metabolism (3, 4). The type and ratio of fatty acids significantly influence their physicochemical properties and biological effects (5). Polyunsaturated fatty acids (PUFAs), which contain two or more double bonds, are categorized into ω-3 and ω-6 PUFAs based on the position of these bonds. As essential fatty acids, they must be obtained through dietary intake to meet physiological requirements (6). Previous studies have demonstrated that supplementation with ω-3 PUFAs can reduce all-cause mortality and the incidence of adverse cardiovascular events in patients at high risk for CVDs (7–9). Furthermore, blood concentrations of ω-3 PUFAs or increased dietary intake have been shown to be inversely associated with the incidence of congestive HF (10, 11). However, recent studies have yielded conflicting results regarding the effects of ω-3 PUFAs on CVDs. Several clinical trials indicate that ω-3 PUFAs supplementation does not significantly reduce the incidence of major adverse cardiovascular events (12–15). Nicholls et al. (13) and Gencer et al. (16) have reported that ω-3 PUFAs supplementation is significantly associated with an increased risk of atrial fibrillation. Similarly, Laguzzi et al. (17) have indicated that low blood levels of eicosapentaenoic acid (EPA) or docosahexaenoic acid (DHA), when accompanied by high concentrations of trans-fatty acids, may elevate the risk of CVDs. Elevated levels of ω-3 PUFAs can alter cell membrane properties and inhibit Na-K-ATPase activity (18). Due to their structural characteristic of containing multiple double bonds, ω-3 PUFAs (particularly DHA) are susceptible to attack by reactive oxygen species (ROS), leading to lipid peroxidation, mitochondrial dysfunction, and DNA damage (19).

Myocardial strain is an advanced imaging biomarker for assessing cardiac function. It quantifies the deformation of myocardial fibers from end-diastole to end-systole, offering greater sensitivity and accuracy than left ventricular ejection fraction (LVEF) in detecting myocardial abnormalities. This technique is capable of identifying both subclinical myocardial dysfunction and segmental or global functional impairments (20). Cardiac magnetic resonance feature tracking (CMR-FT) is an emerging technique for assessing myocardial strain. Utilizing standard steady-state free precession (SSFP) sequences, it enables precise quantitative analysis of both local and global myocardial deformation. As a noninvasive method for evaluating cardiac structure and function, CMR-FT does not require additional image acquisition and offers advantages such as a high signal-to-noise ratio and a wide field of view. These combined strengths contribute to its significant clinical utility (20, 21).

In summary, the impact of ω-3 PUFAs on left cardiac function remains controversial, with limited evidence specifically addressing their effect on left atrial (LA) and left ventricular (LV) myocardial strain. This study aims to investigate the correlation between blood ω-3 PUFAs levels and LA and LV myocardial strain in hypertensive patients using CMR-FT technology. The findings are expected to clarify the influence of ω-3 PUFAs on left cardiac function and provide a basis for the early prevention of left cardiac dysfunction in clinical practice.

## 2 Materials and methods

### 2.1 Patient enrollment

A total of 364 patients who underwent CMR examination between September 2018 and September 2024 with a LVEF≥50% were retrospectively enrolled. CMR images, routine clinical information, and relevant laboratory data were collected for all participants. Clinical information included sex, age, systolic and diastolic blood pressure, medications (antihypertensives, statins, ω-3 supplements), fish intake, physical activity, duration of hypertension, smoking history, and alcohol consumption. Laboratory data included levels of EPA, DHA, docosapentaenoic acid (DPA), alpha-linolenic acid (ALA), total ω-3 PUFAs, low density lipoprotein cholesterol (LDL-C), triglycerides (TG), and total cholesterol (TC). Based on current clinical diagnostic criteria for hypertension, patients were divided into a hypertension group (n = 179) and a control group (n = 185). The hypertension group consisted of 121 males and 58 females, while the control group included 83 males and 102 females.

### 2.2 CMR image acquisition and post-processing

All participants underwent CMR examination on a 3.0T MAGNETOM Skyra scanner (Siemens Healthcare, Erlangen, Germany). Cine imaging was performed using a standard SSFP sequence. Conventional axial, sagittal, and coronal views were acquired to localize cardiac orientation. Subsequent post-processing of the images was conducted to extract quantitative cardiac parameters.

**Figure 1:**
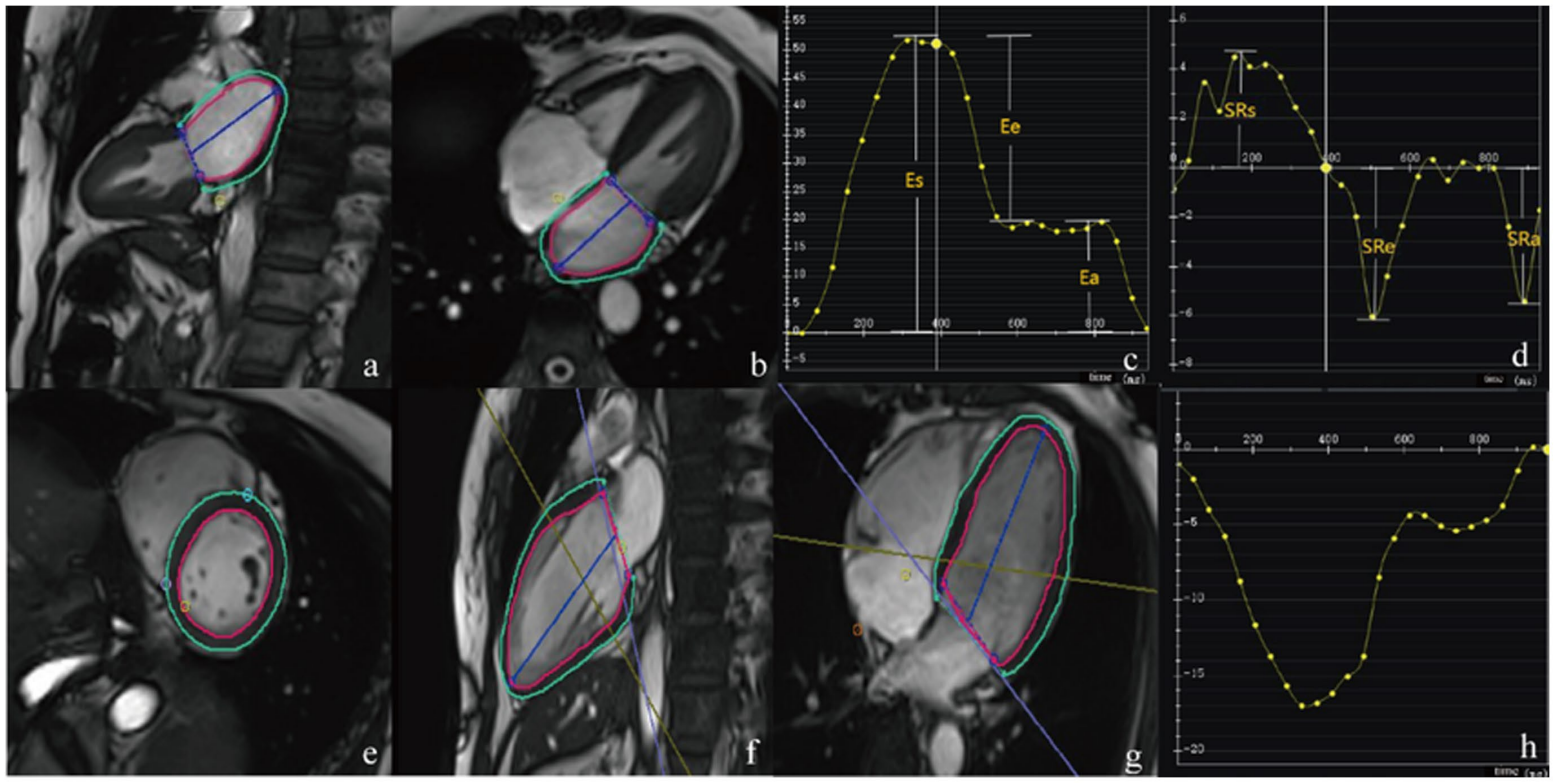
Cardiac magnetic resonance feature tracking and strain curve: LA strain **(a–d)**; LV strain **(e–h)**.

The CMR-FT module of the CVI42 software was used to automatically generate curves depicting LA strain and strain rate throughout the cardiac cycle. The following strain parameters were calculated: total LA strain (LA reservoir strain, LA-Es), passive LA strain (LA conduit strain, LA-Ee), and active LA strain (LA booster strain, LA-Ea). Correspondingly, three strain rate parameters were assessed: peak positive LA strain rate (LA-SRs), peak early negative LA strain rate (LA-SRe), and peak late negative LA strain rate (LA-SRa), as illustrated in Figure 1.

The FT module of the CVI42 software was employed to automatically compute LV global and segmental strain values throughout the cardiac cycle. LV global strain parameters included global longitudinal strain (GLS), global circumferential strain (GCS), and global radial strain (GRS). LV segmental strain comprised longitudinal strain at the apical, mid, and basal levels (LS-apical, LS-mid, LS-basal), radial strain at the apical, mid, and basal levels (RS-apical, RS-mid, RS-basal), and circumferential strain at the apical, mid, and basal levels (CS-apical, CS-mid, CS-basal), as shown in Figure 1.

### 2.3 Statistical analysis

Data normality was assessed using the Shapiro–Wilk test. Continuous variables are presented as mean ± standard deviation (SD) or median (interquartile range, IQR) based on their distribution, while categorical variables are expressed as frequencies. For normally distributed continuous variables, independent-samples *t*-test was applied, for non-normally distributed continuous variables, the Mann–Whitney *U* test was used. The chi-square (*χ*^2^) test was employed for categorical variables. The differences in ω-3 PUFAs levels between the hypertension group and the control group were examined using analysis of covariance (ANCOVA). Univariate and multivariate linear regression was conducted to assess whether ω-3 PUFAs levels served as independent influencing factors on left-sided myocardial strain parameters. Univariate logistic regression was used to analyze the effect of theω-3 on LA and LV myocardial dysfunction. Receiver operating characteristic (ROC) curves were plotted to evaluate the predictive performance of this factor for left-sided myocardial dysfunction, with area under the curve (AUC) determined. All statistical analyses were performed using SPSS 26.0 (SPSS Inc., Chicago, IL, United States) and MedCalc. The graphs were generated with GraphPad Prism 9 and RStudio 4.1.2. Inter-observer consistency for LA and LV myocardial strain parameters was evaluated by calculating the intraclass correlation coefficient (ICC). *p* < 0.05 was considered statistically significant.

## 3 Results

### 3.1 Baseline clinical characteristics

A total of 364 patients who underwent CMR examination at our institution were included in this study. Baseline characteristics of the overall population, the hypertension group, and the control group are summarized in Table 1. According to current clinical diagnostic criteria for hypertension, patients were stratified into a hypertension group (*n* = 179) and a control group (*n* = 185). The hypertension group comprised 121 males and 58 females with a mean age of 56.01 ± 10.00 years, while the control group consisted of 83 males and 102 females with a mean age of 51.90 ± 10.20 years. Significant differences (*p* < 0.05) were observed between the two groups in age (*p* < 0.001), sex (*p* < 0.001), systolic blood pressure (*p* < 0.001), diastolic blood pressure (*p* < 0.001), TG (*p* < 0.001), and creatinine (Cr, *p* = 0.004). In contrast, no significant differences (*p* > 0.05) were found in smoking (*p* = 0.227), alcohol consumption (*p* = 0.640), LDL-C (*p* = 0.750), TC (*p* = 0.439), estimated glomerular filtration rate (eGFR, *p* = 0.850), statins (*p* = 0.439), ω-3 supplements (*p* = 0.874), fish intake (*p* = 0.227), and physical activity (*p* = 0.053).

**Table 1:** Clinical baseline data of the hypertension group and the control group.

Clinical information	Total (*n* = 286)	Hypertension group (*n* = 136)	Control group (*n* = 150)	*P*
Age (year)	53.82 ± 10.0	55.37 ± 9.4	52.42 ± 10.3	0.013
Sex (%)				
Male	160(56)	85(63)	75(50)	0.033
Female	126(44)	51(37)	75(50)	
SBP (mmHg)	134(29)	144(26)	124(24)	<0.001
DBP (mmHg)	88(17)	93(15)	82(13)	<0.001
BMI (kg/m^2^)	25.24(4.81)	26.04(5.45)	24.28(4.56)	<0.001
Smoking (%)	53(18.5)	27(19.8)	26(17.3)	0.584
Drinking (%)	22(7.7)	11(50.0)	11(50.0)	0.811
LDL-C (mmol/L)	3.08(1.11)	3.14(1.26)	3.03(1.02)	0.714
TC (mmol/L)	5.16 ± 1.0	5.16 ± 1.1	5.16 ± 1.0	0.977
TG (mmol/L)	1.45(1.28)	1.79(1.47)	1.21(0.80)	<0.001
Cr (μmol/L)	64.6(19.26)	67.1(20.00)	62.5(18.78)	0.038
eGFR (ml/min)	120.5(43.3)	122.9(48.8)	119.2(36.8)	0.458
HbA1c (mmol/mol)	5.70(0.60)	5.80(0.90)	5.50(0.50)	<0.001
Statins (%)	253(69.5)	118(32.4)	135(37.1)	0.144
ω-3 supplements (%)	151(41.50)	75(20.6)	76(20.9)	0.874
Fish intake (times/week)	2(2)	2(2)	2(2)	0.227
Physical activity (h/week)	2.80(2.40)	3.00(2.40)	2.60(2.20)	0.053

SBP, systolic blood pressure; DBP, diastolic blood pressure; BMI, body mass index; LDL-C, Low-density lipoprotein cholesterol; TC, total cholesterol; TG, triglycerides; Cr, creatinine; eGFR, estimated glomerular filtration rate. HbA1c, HbA1c.glycosylated hemoglobin.

### 3.2 ω-3 PUFAs levels in the hypertension and control groups

Comparisons of ω-3 PUFAs levels between the hypertension and control groups are presented in Table 2. No significant differences were observed in the levels of individual ω-3 PUFA subtypes or total ω-3 between the hypertensive group and the control group (all *p* > 0.05). Specifically, EPA levels were 175.254 ± 12.244 nmoL/mL in the hypertensive group and 176.251 ± 11.917 nmoL/mL in the control group (*p* = 0.964); DHA levels were 593.446 ± 242.893 nmoL/mL and 559.946 ± 241.121 nmoL/mL, respectively (*p* = 0.165); DPA levels were 116.285 ± 73.759 nmoL/mL and 101.930 ± 55.071 nmoL/mL, respectively (*p* = 0.486); *α*-linolenic acid (ALA) levels were 208.151 ± 134.068 nmoL/mL and 159.605 ± 99.342 nmoL/mL, respectively (*p* = 0.470); and total ω-3 levels were 1098.24 ± 708.330 nmoL/mL and 925.993 ± 454.934 nmoL/mL, respectively (*p* = 0.890).

**Table 2:** Comparison of ω-3 PUFAs levels between the hypertensive group and the control group.

Fatty acid	Total (*n* = 364)	Hypertensive group (*n* = 179)	Control group (*n* = 185)	*P*
EPA (nmol/ml)	175.761 ± 128.641	175.254 ± 12.244	176.251 ± 11.917	0.964
DHA (nmol/ml)	576.415 ± 242.241	593.446 ± 242.893	559.946 ± 241.121	0.165
DPA (nmol/ml)	108.989 ± 65.243	116.285 ± 73.759	101.930 ± 55.071	0.486
ALA (nmol/ml)	183.478 ± 120.028	208.151 ± 134.068	159.605 ± 99.342	0.470
Total ω-3 (nmol/ml)	1010.701 ± 598.641	1098.24 ± 708.330	925.993 ± 454.934	0.890

EPA, eicosapentaenoic acid; DHA, docosahexaenoic acid; DPA, docosapentaenoic acid; ALA, α-linolenic acid.

### 3.3 Multivariate linear regression analysis of left cardiac myocardial strain in the hypertension group

**Figure 2:**
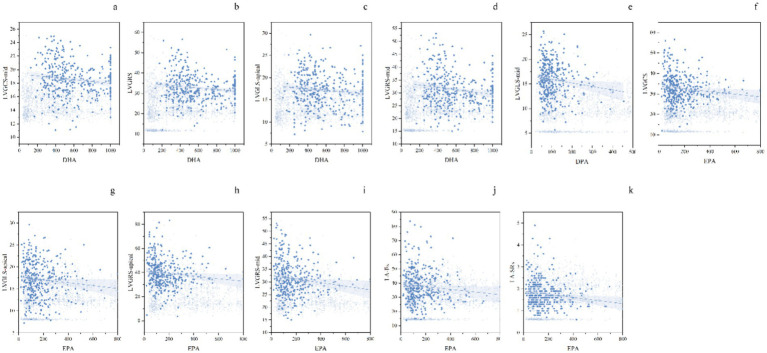
Grouped scatter plots with blue data points and trend lines illustrate the relationships between myocardial strain parameters and ω-3 PUFAs. Figures a–d present the multivariate linear regression results of DHA with LV myocardial strain. Figure e shows the results for DPA with LVGLS-mid. Figures f–k display the multivariate linear regression results of EPA in relation to LA and LV myocardial strain. ω-3 PUFAs, ω-3 polyunsaturated fatty acids; DHA, docosahexaenoic acid; LV, left ventricular; DPA, docosapentaenoic acid; LVGLS-mid, left ventricular global longitudinal strain at mid-wall; EPA, eicosapentaenoic acid; LA, left atrial.

Multivariate linear regression analysis, adjusted for factors that showed statistical significance in univariate analyses, including age, smoking, drinking, body mass index (BMI), Cr, antihypertensives, statins, ω-3 supplements, fish intake, physical activity, and duration of hypertension, revealed the following independent associations (Table 3 and Figure 2). Specifically, DPA was independently and negatively associated with LS-mid (*p* = 0.031); DHA was independently and negatively associated with GRS, LS-apical, RS-mid, and CS-mid (all *p* < 0.05); and EPA was independently and negatively associated with LA-Es, LA-SRs, LS-apical, GRS, RS-mid, and RS-apical (all *p* < 0.05).

**Table 3:** Multivariable linear regression analysis of LA and LV myocardial strain in hypertensive group and its gender subgroups.

ω-3 PUFAs		Total			Male			Female		
		*β*	*t*	*P*	*β*	*t*	*P*	*β*	*t*	*P*
DPA	LS-mid	−0.106	−2.165	0.031						
	RS-basal							−0.169	−2.199	0.029
	CS-basal							−0.158	−2.055	0.042
DHA	GRS	−0.162	−2.499	0.013						
	RS-mid	−0.163	−2.569	0.011						
	RS-basal							−0.171	−2.209	0.029
	RS-apical				−0.151	−2.132	0.034			
	LS-apical	−0.097	−2.013	0.045						
	CS-mid	−0.133	−2.081	0.038						
	CS-basal							−0.155	−2.003	0.047
EPA	GCS	−0.103	−1.999	0.047				−0.159	−2.068	0.040
	GRS							−0.164	−2.062	0.041
	RS-basal							−0.159	−2.068	0.040
	RS-mid	−0.125	−2.644	0.009						
	RS-apical	−0.160	−2.624	0.009						
	LA-Es	−0.142	−2.329	0.021						
	LA-SRs	−0.173	−2.789	0.006						
	LS-apical	−0.112	−2.126	0.034						

ω-3 PUFAs ω-3polyunsaturated fatty acids; EPA eicosapentaenoic acid; DHA docosahexaenoic acid; DPA docosapentaenoic acid; LA left atrial; LA-Es LA reservoir strain; LA-SRs LA positive peak strain rate; LV left ventricular; GLS global longitudinal strain; GRS global radial strain; GCS global circumferential strain.

### 3.4 Sex-stratified multivariate linear regression analysis in the hypertension group

**Figure 3:**
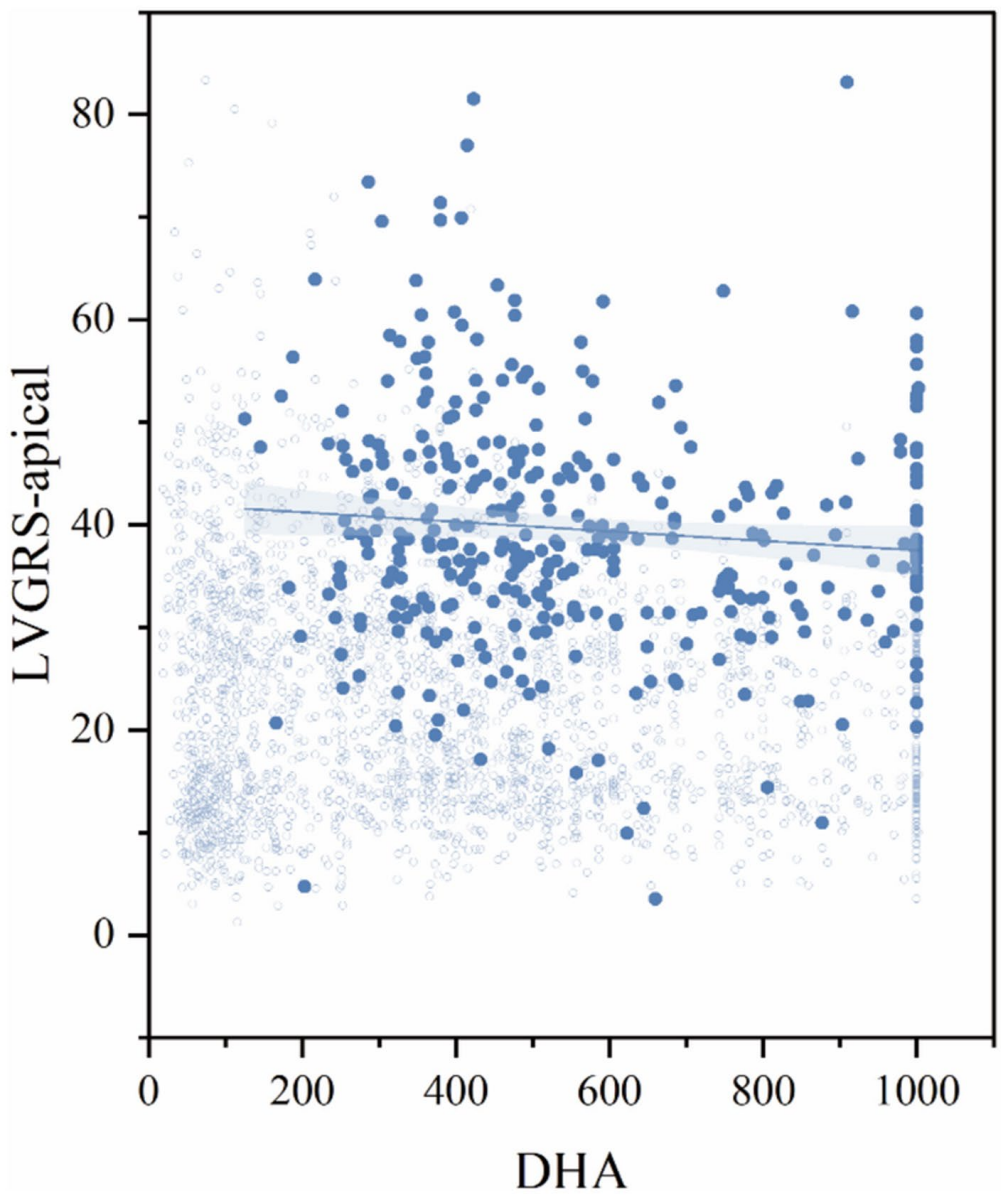
Results of multivariable linear regression for the hypertensive male subgroup.

**Figure 4:**
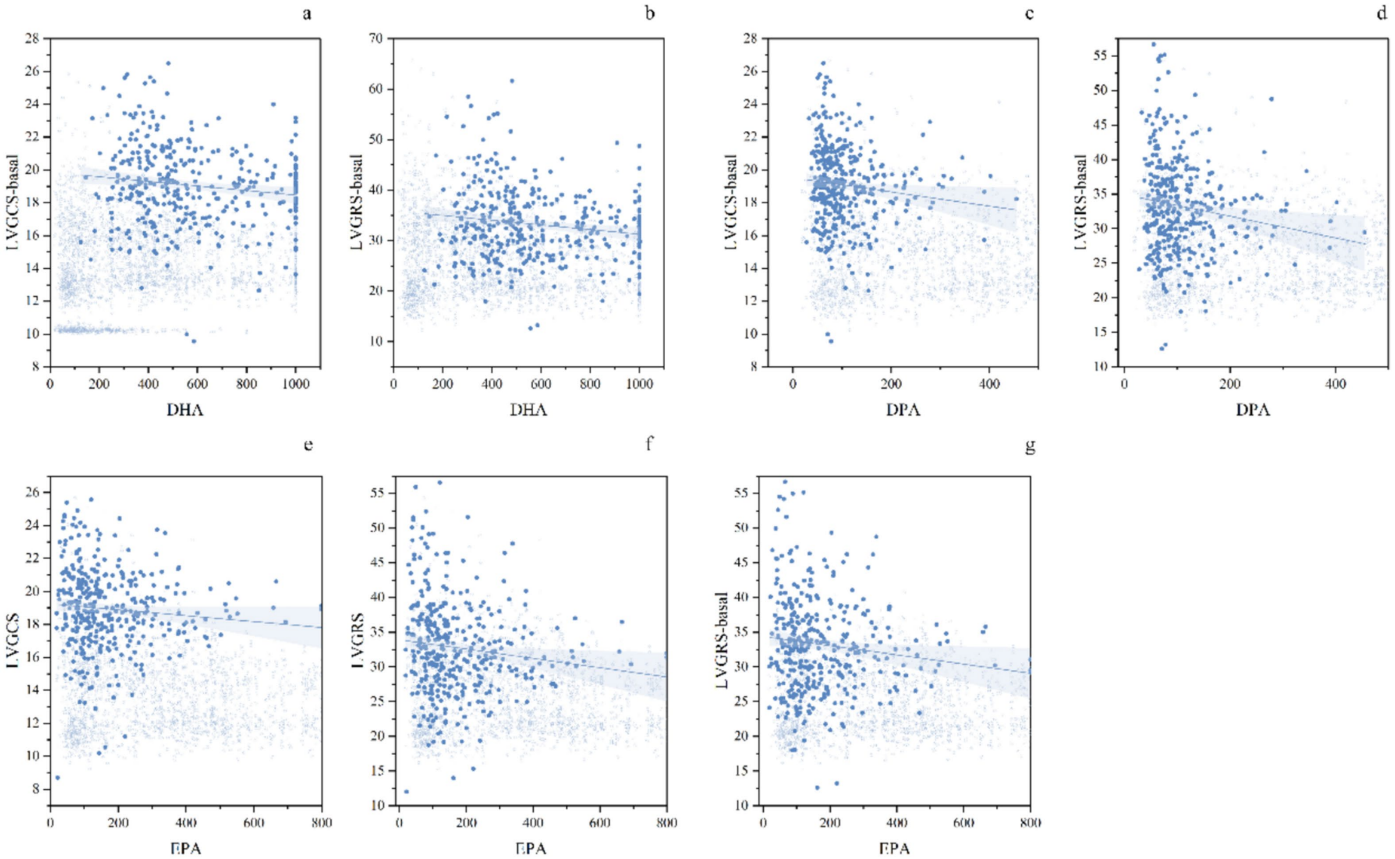
Grouped scatter plots with blue data points and trend lines illustrate the relationships between myocardial strain parameters and ω-3 PUFAs. Figures a–b present the multivariate linear regression results of DHA with LVGCS-basal and LVGRS-basal. Figures c–d show the results for DPA with LVGCS-basal and LVGRS-basal. Figures e–g display the multivariate linear regression results of EPA in relation to LVGCS, LVGRS, and LVGRS-basal. ω-3 PUFAs, omega-3 polyunsaturated fatty acids; DHA, docosahexaenoic acid; LVGCS-basal, left ventricular global circumferential strain at basal; LVGRS-basal, left ventricular global radial strain at basal; DPA, docosapentaenoic acid; EPA, eicosapentaenoic acid; LVGCS, left ventricular global circumferential strain; LVGRS, left ventricular global radial strain.

Sex-stratified analysis revealed distinct patterns of association between ω-3 PUFAs and myocardial strain parameters. Adjusted for factors that showed statistical significance in univariate analyses, including age, smoking, drinking, BMI, Cr, antihypertensives, statins, ω-3 supplements, fish intake, physical activity, and duration of hypertension, sex-subgroup multivariable regression analysis revealed significant sex differences in the associations between ω-3 PUFAs subtypes and left cardiac myocardial strain parameters. In male hypertensive patients, only DHA was independently and negatively associated with RS-apical (*β* = −0.151, *p* = 0.034); in female hypertensive patients, DHA was independently and negatively associated with RS-basal and CS-basal (*β* = −0.171, −0.155, both *p* < 0.05), DPA was independently and negatively associated with RS-basal and CS-basal (*β* = −0.169, −0.158, both *p* < 0.05), and EPA was independently and negatively associated with GRS, RS-basal, and GCS (*β* = −0.164, −0.159, −0.159, all *p* < 0.05) (show in Table 3 and Figures 3, 4).

### 3.5 Logistic regression analysis of the influence of ω-3 PUFAs and subtypes on left cardiac myocardial dysfunction

Based on previous studies defining LA reservoir, conduit, and booster strain averages as 34.9, 21.3, and 14.3% respectively (22), and GLS < 16% as indicative of early LV myocardial dysfunction (23), we defined LA-Es < 35% and GLS < 16% as thresholds for LA and LV myocardial dysfunction, respectively. Univariate logistic regression analysis was performed with ω-3 PUFAs and subtypes as the independent variable and dichotomized GLS (<16% vs. ≥16%) or LA-Es (<35% vs. ≥35%) as the dependent variable.

As shown in Tables 4, 5, Univariate logistic regression analysis showed that certain ω-3 PUFA subtypes and total ω-3 levels were significantly associated with LA and LV myocardial dysfunction in hypertensive patients. For LA dysfunction, DHA (OR = 1.002, *p* = 0.010), EPA (OR = 1.003, *p* = 0.009), and total ω-3 (OR = 1.001, *p* = 0.037) were associated with increased risk, while DPA and ALA showed no significant associations (both *p* > 0.05). For LV myocardial dysfunction, DHA (OR = 1.002, *p* < 0.001), DPA (OR = 1.005, *p* = 0.020), and total ω-3 (OR = 1.001, *p* < 0.001) were associated with increased risk, whereas EPA and ALA showed no significant associations (both *p* > 0.05). It showed that no significant associations were observed between individual ω-3 PUFA subtypes or total ω-3 levels and LA or LV myocardial dysfunction in the control group (all *p* > 0.05).

**Table 4:** Univariate logistic regression analysis of the impact of the ω-3 PUFAs on LA and LV myocardial dysfunction in hypertensive groups.

ω-3 PUFAs	LA			LV	
	OR 95%CI		*P*	OR 95%CI	*P*
DHA	1.002	1.000–1.003	0.010	1.002 1.000–1.003	0.012
DPA	1.003	1.000–1.007	0.199	1.005 1.001–1.009	0.020
EPA	1.003	1.001–1.005	0.009	1.001 1.000–1.003	0.192
ALA	1.002	1.000–1.005	0.085	1.002 1.000–1.004	0.184
Total ω-3	1.001	1.000–1.001	0.037	1.001 1.000–1.001	0.053

ω-3 PUFAs ω-3polyunsaturated fatty acids; EPA eicosapentaenoic acid; DHA docosahexaenoic acid; DPA docosapentaenoic acid; ALA α-linolenic acid; LA left atrial; LV left ventricular; OR odds ratio; CI confidence interval.

**Table 5:** Univariate logistic regression analysis of the impact of the ω-3 PUFAs on LA and LV myocardial dysfunction in control groups.

ω-3 PUFAs	LA			LV	
	OR 95%CI		*P*	OR 95%CI	*P*
DHA	0.999	1.000–1.001	0.334	1.001 1.000–1.003	0.147
DPA	0.997	1.000–1.003	0.369	1.002 1.000–1.009	0.627
EPA	1.001	1.000–1.003	0.681	1.001 1.000–1.005	0.461
ALA	1.001	1.000–1.003	0.921	1.002 1.000–1.005	0.280
Total ω-3	1.001	1.000–1.001	0.301	1.001 1.000–1.001	0.218

ω-3 PUFAs ω-3polyunsaturated fatty acids; EPA eicosapentaenoic acid; DHA docosahexaenoic acid; DPA docosapentaenoic acid; ALA α-linolenic acid; LA left atrial; LV left ventricular; OR odds ratio; CI confidence interval.

### 3.6 ROC analysis of ω-3 PUFAs and subtypes for predicting left cardiac myocardial dysfunction in hypertension

**Figure 5:**
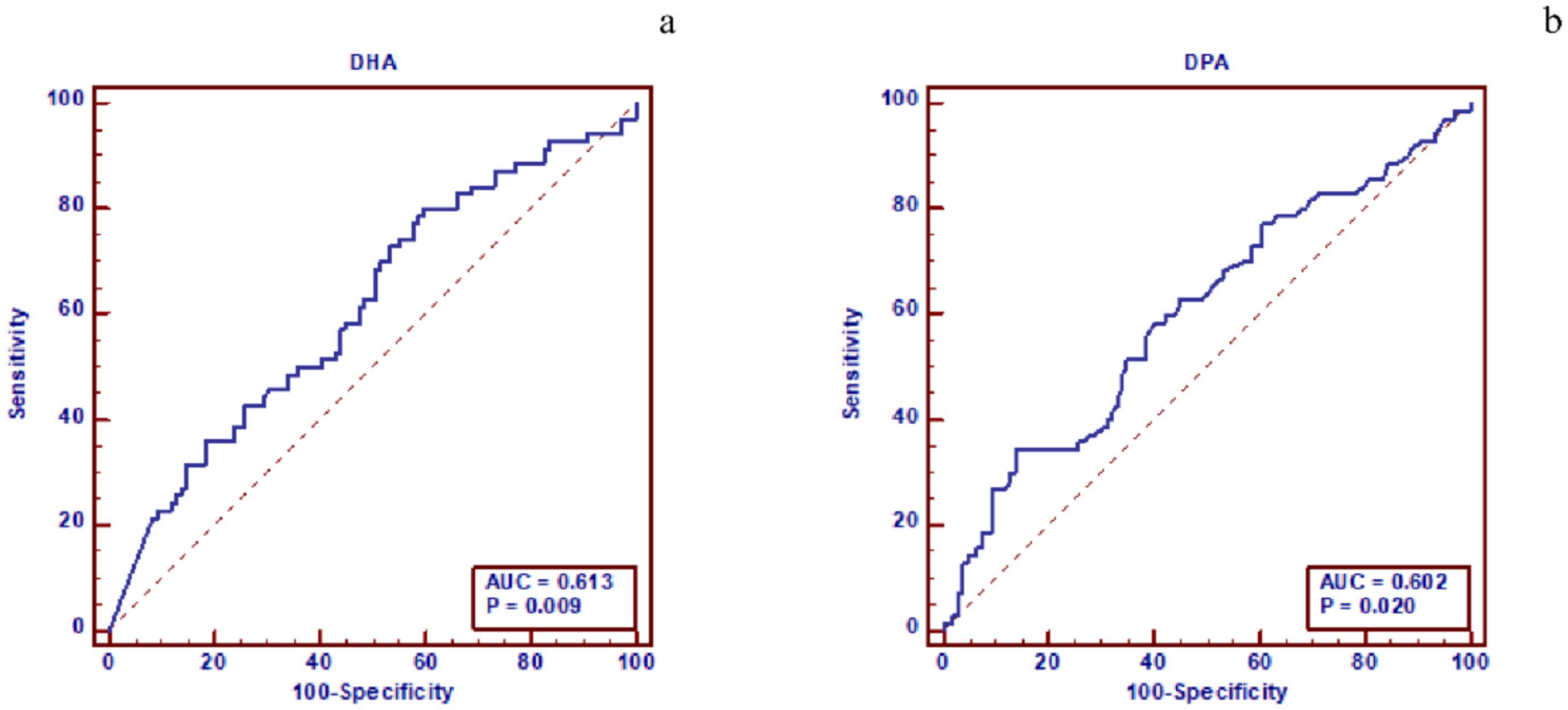
Figure **(a)** shows the predictive performance of DHA for LV dysfunction in hypertensive patients. The ROC curve indicates that the area under the curve (AUC) of DHA for predicting LV dysfunction is 0.613, with a P value of 0.009. Figure **(b)** shows the predictive performance of DPA for LV dysfunction in hypertensive patients. The ROC curve indicates that the AUC of DPA for predicting LV dysfunction is 0.602, with a P value of 0.020. DHA, docosahexaenoic acid; ROC, receiver operating characteristic; AUC, area under the curve; DPA, docosapentaenoic acid; LV, left ventricular.

**Figure 6:**
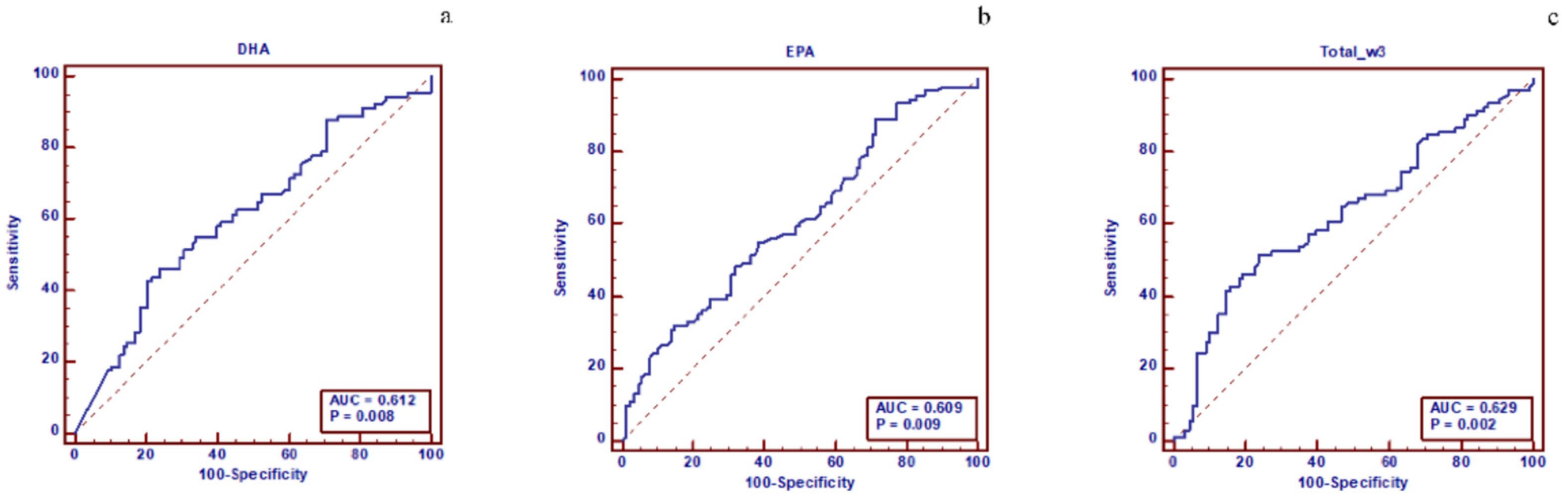
Figure **(a)** shows the predictive performance of DHA for LA dysfunction in hypertensive patients. The ROC curve indicates that the area under the curve (AUC) of DHA for predicting LA dysfunction is 0.612, with a P value of 0.008. Figure **(b)** shows the predictive performance of EPA for LA dysfunction in hypertensive patients. The ROC curve indicates that the AUC of EPA for predicting left atrial dysfunction is 0.609, with a P value of 0.009. Figure **(c)** shows the predictive performance of total ω-3 PUFAs for LA dysfunction in hypertensive patients. The ROC curve indicates that the AUC of total ω-3 PUFAs for predicting LA dysfunction is 0.629, with a P value of 0.002. Abbreviations: ω-3 PUFAs, omega-3 polyunsaturated fatty acids; DHA, docosahexaenoic acid; EPA, eicosapentaenoic acid; ROC, receiver operating characteristic; AUC, area under the curve; LA, left atrial.

The ROC curve analysis showed that both DHA and DPA had moderate predictive value for LV myocardial dysfunction in hypertensive patients, with an area under the curve (AUC) of 0.613 (95% CI: 0.538–0.685), sensitivity of 80.00%, specificity of 40.37%, and Youden index of 0.204 for DHA, and an AUC of 0.602 (95% CI: 0.555–0.658), sensitivity of 34.29%, specificity of 86.34%, and Youden index of 0.205 for DPA. Additionally, DHA, EPA, and total ω-3 PUFAs demonstrated moderate predictive value for LA myocardial dysfunction in hypertensive patients, with an AUC of 0.612 (95% CI: 0.537–0.684), sensitivity of 42.86%, specificity of 79.55%, and Youden index of 0.224 for DHA; an AUC of 0.609 (95% CI: 0.534–0.681), sensitivity of 89.01%, specificity of 28.41%, and Youden index of 0.174 for EPA; and an AUC of 0.629 (95% CI: 0.554–0.770), sensitivity of 51.65%, specificity of 76.14%, and Youden index of 0.278 for total ω-3 (show in Tables 6, 7 and Figures 5, 6).

**Table 6:** ROC analysis of the prediction model for LV myocardial dysfunction in hypertensive groups.

ω-3 PUFAs	AUC (95%CI)	Sensitivity (%)	Specificity (%)	Youden index
DHA	0.613(0.538–0.685)	80.00	40.37	0.204
DPA	0.602(0.555–0.658)	34.29	86.34	0.205

ω-3 PUFAs ω-3polyunsaturated fatty acids; DHA docosahexaenoic acid; DPA docosapentaenoic acid; LV left ventricular; ROC receiver operating characteristic; AUC area under the curve.

**Table 7:** ROC analysis of the prediction model for LA myocardial dysfunction in hypertensive groups.

ω-3 PUFAs	AUC (95%CI)	Sensitivity (%)	Specificity (%)	Youden index
DHA	0.612(0.537–0.684)	42.86	79.55	0.224
EPA	0.609(0.534–0.681)	89.01	28.41	0.174
Total ω-3	0.629(0.554–0.770)	51.65	76.14	0.278

ω-3 PUFAs ω-3polyunsaturated fatty acids; DHA docosahexaenoic acid; EPA eicosapentaenoic acid; LA left atrial; ROC receiver operating characteristic; AUC area under the curve.

### 3.7 Inter-observer consistency analysis of LA and LV myocardial strain parameters

To assess inter-observer consistency, two radiologists independently analyzed LA and LV myocardial strain parameters in a randomly selected subset of 90 patients from the hypertension group. Both observers used CVI42 software to contour the myocardium and derive strain parameters, including LA-Es, LA-Ee, LA-Ea, GLS, GRS, and GCS. The inter-observer agreement for all measured parameters was good, as detailed in Table 8.

**Table 8:** Inter-observer agreement analysis for LA and LV myocardial strain parameters.

Variable	Inter-Observer	
	ICC	95%CI
LA-Es	0.987	0.980–0.991
LA-Ea	0.998	0.997–0.999
LA-Ee	0.978	0.967–0.985
GLS	0.896	0.846–0.930
GRS	0.984	0.976–0.989
GCS	0.898	0.849–0.931

ICC, intraclass correlation coefficient; LA-Es, LA reservoir strain; LA-Ea, LA booster strain; LA-Ee, LA conduit strain; GLS, global longitudinal strain; GRS, global radial strain; GCS, global circumferential strain; CI, confidence interval.

## 4 Discussion

The impact of ω-3 PUFAs on left cardiac function remains a subject of ongoing debate. Several trials have highlighted conflicting results regarding their effects on cardiovascular function. For instance, Chen et al. (12) reported that regular use of fish oil supplements was associated with an increased relative risk of stroke in individuals without pre-existing CVDs. A study demonstrated that daily supplementation with ω-3 PUFAs did not significantly reduce the risk of major adverse cardiovascular events in diabetic patients without evidence of CVDs (14). Myocardial strain imaging has been demonstrated to effectively detect early cardiac dysfunction in various CVDs. As a precise imaging modality for assessing myocardial strain, CMR-FT offers unique advantages in evaluating both global and segmental myocardial function (21, 24). Currently, there is limited research on the effects of ω-3 PUFAs on left cardiac myocardial strain. In this study, we first compared ω-3 PUFAs levels between the hypertensive group and the control group. The results showed no significant differences in the levels of EPA, DHA, DPA, ALA, or total ω-3 between the two groups (all *p* > 0.05). This finding holds important implications. First, it eliminates the potential confounding effect of baseline differences in fatty acid levels between the two groups on subsequent association analyses, ensuring that the negative association observed between ω-3 PUFAs and myocardial strain within the hypertensive group more accurately reflects disease-specific effects rather than intergroup baseline differences. Second, previous studies have indicated that hypertensive patients often exhibit fatty acid metabolism disorders, including reduced fatty acid oxidation capacity and lipid accumulation (25). However, the comparable ω-3 PUFAs levels between the two groups in this study suggest that the hypertensive patients in this cohort may be in the early stages of the disease or in a state of metabolic compensation, without evident abnormalities in ω-3 PUFAs levels. Furthermore, there were no significant differences between the two groups in lifestyle factors such as fish intake, ω-3 supplement, and physical activity (all *p* > 0.05), indicating that the two populations may have similar dietary structures, fish consumption frequency, and supplement usage, which likely contributed to the lack of significant differentiation in fatty acid levels (26), further supporting the comparability of fatty acid levels between the two groups.

Myocardial strain, as a sensitive indicator reflecting the deformation capacity of myocardial fibers from end-diastole to end-systole, depends on adequate energy supply and intact cellular structure for its maintenance. In this study, EPA, DHA, and DPA were independently and negatively correlated with multiple left cardiac myocardial strain parameters, suggesting that higher ω-3 PUFAs levels correspond to poorer myocardial strain capacity. The discrepancy between these findings and previous understandings can be explained from the following aspects. First, the effects of ω-3 PUFAs are dose-dependent. Animal experiments and *in vitro* studies have shown that low concentrations of ω-3 PUFAs can improve myocardial energy metabolism, whereas high concentrations can induce lipid peroxidation and mitochondrial dysfunction (27) Gencer et al. (16) reported that daily supplementation with more than 1 gram of ω-3 PUFAs is significantly associated with an increased risk of atrial fibrillation (28). In this study, the mean DHA level in the hypertension group was approximately 593 nmoL/mL, equivalent to about 0.59 μmol/L. Although this is lower than the previously reported threshold for Na-K-ATPase inhibition (3.75 μmol/L) (29), considering the accumulation effect of ω-3 PUFAs in myocardial tissue and individual variability, some patients may have reached the biological effect threshold. Second, ω-3 PUFAs, particularly DHA and EPA, possess multiple double bonds that confer chemical instability, rendering them susceptible to attack by reactive oxygen species (ROS) and subsequent initiation of lipid peroxidation (19). DHA contains six double bonds, making it a highly PUFA that is extremely susceptible to peroxidative attack (30). Furthermore, ω-3 PUFAs can promote mitochondrial dysfunction and initiate lipid peroxidation, which reduces ATP generation. As a high-energy-consuming chamber, the global systolic function (GRS, GCS) and segmental systolic function (RS-mid, LS-apical, CS-mid) of the LV are extremely sensitive to energy supply, with energy metabolism disorders directly manifesting as reduced strain capacity. Second, alterations in the physicochemical properties of the cell membrane have important implications for left atrial function. Atrial cardiomyocytes possess a high density of ion channels (e.g., calcium channels, potassium channels), whose function is highly dependent on the membrane phospholipid microenvironment. When EPA is incorporated into the cell membrane, it can modify membrane fluidity and lipid raft structure, thereby affecting calcium channel function and calcium transients (31). The LA-SRs directly reflects the active contractile capacity of the myocardium, and its reduction is closely associated with abnormal calcium handling. These ultimately lead to myocardial damage and functional decline. On the other hand, Laguzzi et al. (17) noted that low blood levels of EPA or DHA may coexist with elevated concentrations of trans-fatty acids. Trans-fatty acids have been shown to significantly elevate levels of LDL-C and very-low-density lipoprotein (VLDL), while reducing HDL-C. They also induce oxidative stress, cause damage to vascular endothelial cells, and thereby promote the development of atherosclerosis, ultimately increasing the risk of CVDs (32).

The present study also revealed that ω-3 PUFAs have a more pronounced impact on left cardiac myocardial strain in female hypertensive patients compared to their male counterparts. This may be because estrogen influences fatty acid uptake, oxidation, and accumulation by regulating the expression and activity of enzymes involved in fatty acid metabolism. Estrogen receptor *α* is highly expressed in cardiomyocytes and can modulate the activity of peroxisome proliferator-activated receptors (PPAR) and carnitine palmitoyltransferase 1 (CPT1), thereby affecting fatty acid oxidation efficiency. Second, premenopausal women have higher estrogen levels, which may partially counteract oxidative stress damage through antioxidant and anti-inflammatory mechanisms. However, when ω-3 PUFAs levels increase to a certain extent, their pro-oxidative effects may surpass the protective effects of estrogen, resulting in a double-hit effect (33). Furthermore, studies suggest that female cardiomyocytes are more dependent on fatty acids than male cardiomyocytes, making fatty acid metabolism disorders more likely to induce myocardial dysfunction in women. From a clinical perspective, these findings suggest that sex-specific strategies should be considered in cardiovascular risk management for hypertensive patients, with more caution exercised when recommending ω-3 supplementation in female patients.

The ROC curve analysis demonstrated that different ω-3 PUFA subtypes have moderate predictive value for LA and LV myocardial dysfunction, exhibiting significant organ specificity and subtype specificity in terms of sensitivity and specificity. The LV dysfunction is primarily associated with DHA and DPA, while the LA dysfunction is primarily associated with EPA and total ω-3, with DHA also showing a correlation with LA dysfunction. The LV has a large myocardial mass and high oxygen consumption, and its function is highly dependent on the stability of mitochondrial energy metabolism and the efficiency of the electron transport chain (34). As a key component of mitochondrial membrane phospholipids, DHA has the most direct impact on mitochondrial function when its levels change. In the oxidative stress environment characteristic of hypertension, DHA is prone to peroxidation, generating toxic aldehydes such as 4-hydroxyhexenal. These aldehydes can covalently modify subunits of the respiratory chain complexes, inhibit electron transport chain activity, and reduce ATP synthesis efficiency (27, 35). The left ventricle’s dependence on energy supply makes it highly sensitive to changes in DHA levels, which is reflected in the high sensitivity of DHA in LV dysfunction. DPA exhibits high specificity in LV dysfunction, which may be attributed to its selective distribution in LV myocardial tissue and its unique role in regulating membrane microdomains (36). The LA has a thin wall and strong reserve capacity, rendering it more sensitive to inflammation and local energy metabolism disturbances. As a precursor of anti-inflammatory lipid mediators such as resolvin E1 and protectin D1, EPA plays a key role in regulating inflammatory responses (37). However, under the pathological condition of hypertension, the production of reactive oxygen species is increased (38), and the metabolic balance of EPA may be altered—its conversion to anti-inflammatory mediators becomes less efficient, while its propensity to act as a substrate for peroxidation increases (33). Consequently, elevated EPA levels are associated with a widespread increase in the risk of LA dysfunction, reflecting the high sensitivity of EPA in LA dysfunction.

It is noteworthy that different ω-3 PUFA subtypes exhibit complementary characteristics in sensitivity and specificity when predicting left cardiac dysfunction, a phenomenon with important mechanistic implications. At the LV level, the high sensitivity of DHA stems from its highly unsaturated structure and widespread distribution in cardiomyocytes. Its peroxidation products can activate the mitochondrial permeability transition pore, induce loss of mitochondrial membrane potential and cytochrome c release, and initiate cardiomyocyte apoptosis cascades (33, 39). This broad-spectrum damage mechanism results in a widespread increase in the risk of LV dysfunction when DHA levels are elevated. In contrast, the high specificity of DPA is associated with its more localized targets. DPA undergoes selective accumulation in myocardial tissue and can preferentially integrate into specific membrane microdomains, affecting local signal transduction and ion channel function (40), making the association between elevated DPA levels and LV dysfunction more specific. At the LA level, the high sensitivity of EPA is related to its key role in the inflammation-oxidative stress balance in the LA. The LA is highly sensitive to inflammatory responses, and alterations in EPA metabolic balance can rapidly affect the local inflammatory microenvironment of the LA. Meanwhile, EPA can undergo lipid peroxidation in an oxidative stress environment, generating toxic aldehydes that damage LA cardiomyocytes (35). The high specificity of DHA may stem from the relatively low DHA content in the membrane phospholipid composition of LA cardiomyocytes, requiring DHA levels to reach a higher threshold to exert significant functional effects on the LA (41).

Furthermore, the AUC of total ω-3 for LA dysfunction was 0.629, slightly higher than that of individual subtypes, suggesting that total ω-3 levels may be superior to single subtypes in predicting LA dysfunction, a finding potentially attributable to synergistic effects among different subtypes (26). Notably, all analyses in this study were conducted in hypertensive patients, and no significant association between ω-3 PUFAs and left cardiac dysfunction was observed in the control group, further confirming that the hypertensive state is a key contextual factor driving the transition of ω-3 PUFAs from “protective” substances to “pro-damage” substrates. Under hypertensive conditions, elevated angiotensin II levels activate NADPH oxidase, leading to a marked increase in reactive oxygen species production (38). This oxidative stress environment provides the necessary conditions for ω-3 PUFAs peroxidation. When ω-3 PUFAs levels rise, their total load as peroxidation substrates increases, resulting in the generation of substantial amounts of toxic aldehydes under the action of reactive oxygen species, thereby impairing cardiomyocyte function (27).

This study has several limitations. First, it is a retrospective, single-center study with a relatively single source of samples, which may introduce selection bias. Second, the variety of post-processing software available for CMR-FT leads to considerable inter-study variability in strain measurements and the absence of a unified reference standard; further research is needed to establish standardized criteria. Third, this study did not provide detailed quantification of dietary sources of ω-3 PUAFs.

## 5 Conclusion

This study found that blood ω-3 PUFAs levels in hypertensive patients are associated with impaired left cardiac myocardial strain and an increased risk of myocardial dysfunction, with significant sex differences and subtype specificity. Enhanced oxidative stress in the context of hypertension serves as a key background factor underlying this association. Different ω-3 PUFA subtypes exhibit complementary characteristics in sensitivity and specificity for predicting left cardiac dysfunction, and their combined application may improve predictive efficacy. This study provides a novel combination of biomarkers for early risk assessment of left cardiac dysfunction in hypertensive patients, suggesting that in clinical practice, distinct ω-3 PUFA subtypes should be differentiated, blind supplementation with ω-3 should be avoided, and sex-specific management strategies should be considered.

## Data Availability

The raw data supporting the conclusions of this article will be made available by the authors, without undue reservation.
